# Mindfulness-based cognitive therapy combined with repetitive transracial magnetic stimulation (rTMS) on information processing and working memory of patients with multiple sclerosis

**DOI:** 10.22088/cjim.13.3.607

**Published:** 2022

**Authors:** Majid Eydi-Baygi, Abdolaziz Aflakseir, Mehdi Imani, Mohammad Ali Goodarzi, Mohammad Hossein Harirchian

**Affiliations:** 1 Department of Clinical Psychology, Faculty of Psychology and Education Sciences, Shiraz University, Shiraz, Iran; 2 Iranian Center of Neurological Research, Neuroscience Institute, Imam Khomeini Hospital, Tehran University of Medical Sciences, Tehran, Iran

**Keywords:** Mindfulness-based cognitive therapy, Repetitive transracial magnetic stimulation, Information processing, Working memory, MS patients

## Abstract

**Background:**

MS is a demyelinating disease that can result in significant disability. Along with physical complications, this disease is associated with significant psychological complications, including cognitive decline. Therefore, this study aimed to determine the efficacy of mindfulness-based cognitive therapy in combination with rTMS on information processing and working memory in patients with MS.

**Methods:**

The current study used a single-case experimental design and included a follow-up (A-B-A). The statistical population of the present study was all MS patients in Tehran who referred to Imam Khomeini Hospital in Tehran in 2020. The present study sample consisted of 5 MS patients selected by the sampling methods available**.** Subjects were assessed three times before, during, and after the intervention using the Zahlen-Verbindongs and n-back tests in the two-back position. Subjects received cognitive therapy based on mindfulness and rTMS at a frequency of 10 Hz. Visual and graphical recovery percentage and effect size methods were used to analyze the data.

**Results:**

The current study's findings indicate that combining mindfulness with rTMS has a beneficial effect on the information processing and working memory of MS patients. Overall, 67.24% recovered following the intervention stage, 53.64% recovered following the follow-up for information processing, 104.04% recovered following the intervention stage, and 76.98% recovered following the follow-up for working memory.

**Conclusion:**

The study shows the effect of mindfulness combined with rTMS on cognitive problems in MS patients. Significant improvements in MS patients' information processing, working memory, and therapeutic outcomes were observed throughout the follow-up period.

MS is the most common chronic inflammatory disease of the central nervous system, affecting more than 2 million people worldwide (1). In Iran, 578 out of every 100,000 people are infected with this disease (2). MS mainly affects younger adults with an average age of 30 years. Women are three times as likely to be affected as men (3). The long-term prognosis for this disease is poor; approximately half of the patients require permanent wheelchair use 25 years after diagnosis (4). The exact etiology of multiple sclerosis is still unknown, but autoimmunity, genetics, and environmental factors play an essential role in its development (5, 6). Because MS is a central nervous system disease, the symptoms are not homogeneous and vary per patient. These symptoms are based on neurology, and depending on the location of the lesion, can affect the sensory, motor, visual, and brainstem pathways (4, 7).

The prevalence of cognitive impairment in MS patients is reported to be between 40 and 70% (8-12). The prevalence of cognitive impairment in patients with relapsing-remitting MS (RRMS) is about 30% (13-14). Cognitive areas most often affected by the disease include memory (verbal and work), information processing speed, executive functions, attention, abstract/conceptual reasoning, and spatial skills (14-17). Recent research has demonstrated the critical role that impaired information processing speed plays in learning and memory deficits. In fact, learning new information, encryption, and final retrieval is highly dependent on attention, working memory, and processing speed (18). Additionally, certain findings indicate that working memory, learning, episodic memory, performance, and processing speed are interdependent (19). 

As a result, it is challenging to disentangle the distinct contributions of these distinct cognitive domains to the overall cognitive impairment associated with MS (18). Grigsby et al. (20) observed defects in the prefrontal cortex of these patients. According to their study, problems with central information processing may be the primary cause of diminished cognitive function. Additionally, increasing the amount of time spent processing information from cognitive activities (including memory) can help some of these patients improve their cognitive functions (21). An essential function of the dorsolateral prefrontal cortex includes executive functions such as working memory, cognitive flexibility (22), planning, restraint, and abstract reasoning (23). Drugs used to treat the disease have little effect on cognitive function (24). The cognitive decline appears to persist in a small proportion of patients even after attaining the desired condition with no evidence of disease (25). The role of cognitive rehabilitation in various central nervous system diseases and MS has only recently become apparent (26). 

Additionally, non-physiological interventions are discussed as potentially beneficial in improving the disease's physical and cognitive aspects (27). Experimental approaches using mindfulness-based interventions are appropriate for MS patients with cognitive impairment. Recent studies have shown that mindfulness-based interventions are a promising choice in treating psychological function in MS patients (28-29). Additionally, research indicates that mindfulness meditation can be used therapeutically to improve working memory capacity (30-33). Furthermore, it has been demonstrated that rTMS can alter neurons' structural, functional, and molecular properties, which may be dependent on the simultaneous transfer of action potentials (34-35). Studies have shown that rTMS in combination with medication significantly improves spasm (36-39), fatigue and depression (27), urinary dysfunction (36), gait (40), and agility (41-42) in patients with MS.

In a study, Pradhan et al. (43) showed that the active frequency (10 Hz) of rTMS applied to the left dorsolateral prefrontal cortex combined with meditation practice during rTMS sessions could simultaneously improve attention, cognitive function and reduce the effects of negative reminders on the individual. Thus, the purpose of this study is to determine whether mindfulness-based cognitive therapy in conjunction with rTMS affects the information processing and working memory of MS patients.

## Methods

**Study design:** The current study used a single-case experimental design and included a follow-up (A-B-A). The current study's statistical population included all MS patients referred to Tehran's Imam Khomeini Hospital in 2020. The study sample consisted of 5 MS patients selected by available sampling methods based on inclusion and exclusion criteria. The inclusion criteria included having MS with the approval of a neurologist, having relapsing-remitting MS, providing informed consent to participate in the study, being between the ages of 20 and 45 years, and possessing at least a diploma. Additionally, exclusion criteria included pregnancy and epilepsy, metal prosthetics, cranial shunting, having a pacemaker, refusing to cooperate with treatment sessions for at least two consecutive weeks, and using another type of psychological therapy during the study. Sample selection based on sample entry and exit criteria began in November 2020 and lasted until January 2021. After interviewing the patients with memory problems and explaining the research process, they signed an informed consent form if they agreed to participate in the study. Then, subjects were evaluated by the Zahlen-Verbindongs test and the n-back test in the 2-back position. The subjects were evaluated 4 times at this stage, including the initial evaluation. In stage B, cognitive therapy of mindfulness and treatment of rTMS with a frequency of 10 Hz was performed. The mindfulness treatment was administered once every eight weeks and weekly. Following three sessions of mindfulness, rTMS at a frequency of 10 Hz was performed for ten consecutive sessions. Mindfulness exercises were performed for 15 minutes before rTMS sessions. Subjects were assessed during intervention phase (B) at the end of sessions 2, 5, and 8 of the mindfulness therapy. After the interventions, the subjects were re-evaluated in the follow-up stage (A) twice and once a month.

**Research instrument**

**Zahlen–Verbindungs-Test: **Oswald and Roth devised this test in 1978 (44). The ZVT provides a very reasonable measure of information processing speed and has a high correlation with standard psychometric tests of intelligence (45). This test is an attempt-based test in which subjects must draw lines that connect the numbers 1 to 90, which are very random or, in some cases side by side, on a piece of paper. Subjects are trained to complete the test as quickly and accurately as possible. A study between the performance in ZVT plus the intelligence factor g resulted in a correlation coefficient of 0.62 to 0.77 (45). Completing ZVT necessitates the inclusion of motor and pre-motor processing components. The reliability of this instrument was reported to be 0.86 when using the test-retest method (46).

**N-back test: **This test was first designed and used by Kirchner in 1958 (47). One objective is to assess cognitive function as it pertains to executive actions. Since both data storage and manipulation are required in this test, its application for measuring working memory is considered very appropriate. In this test, several visual stimuli with a distance of 1800 milliseconds appeared on the screen as a chain, and the subject should compare each stimulus to the previous stimulus and press the appropriate key if they are similar (48). The n-back test has a formal validity as a working memory test because it seems to require retention, constant updating, and information processing. Since at least two tasks, information storage and manipulation, must be performed concurrently; it meets the criteria for public domain attention (49). In a study to evaluate the validity of the n-back test, the score of this test was correlated with the combined score obtained from the scores of four complex expansions, including operational, reading, symmetry, and rotation expansiveness, with a correlation of r = 0.55 (50). In a study conducted on 123 students at the University of Illinois America, the reliability coefficient obtained through Cronbach's alpha coefficient ranged from 0.54 to 0.84, which shows the test's high reliability (51).

**Intervention sessions: **The current study included two components: cognitive therapy with mindfulness and rTMS in combination. The mindfulness cognitive therapy program included eight 90-minute sessions scheduled once a week. The program's implementation and training sessions were based on the mindfulness cognitive therapy protocol described in the book Full Catastrophe Living (52).

**Table 1:** Summary of mindfulness sessions

Sessions	Description
**First session**	Communicating and conceptualizing, the importance of mindfulness and familiarity with relaxation, as well as a variety of mindfulness and psychological techniques and practices centered on breathing
**Second session**	Check the tasks of the previous session and the obstacles to its implementation, providing explanations to overcome the obstacles, relaxation training for 14 muscle groups, dealing with sources of tension, concentration, body examination, mindfulness in daily life, presenting homework for the next session (exercise body examination, practice breathing with the presence of mind, record pleasant events every day and record homework reports)
**Third session**	Check homework, relaxation training for 6 muscle groups, doing yoga techniques and presence of mind from breathing, presenting homework for the next session (relaxation exercise, body check exercise, walking with the presence of mind, preparing a list of unpleasant events every day, practice breathing space with the presence of mind, record homework, practice the first part of yoga and practice meditation)
**Fourth session**	Assess homework, perform meditation techniques, present homework for the next session (practice sitting meditation, practicing breathing space, recording homework report, and 20 minutes of mindful breathing before bed)
**Fifth meeting**	Check homework, practicing the presence of experience without judgment, teaching body inspection techniques, including teaching the technique of paying attention to body movement when breathing, focusing on body parts and their movements, and searching for physical senses (hearing, taste, and others), presenting homework for the future (sitting meditation practice, practicing short breathing continuously during the day, recording homework reports and practicing yoga)
**Sixth Session**	Check homework, teaching mindfulness of thoughts including paying attention to the mind, positive and negative thoughts, pleasant or unpleasant thoughts, allowing negative and positive thoughts to enter the mind and quickly taking them out of mind without judgment and focused attention, plus homework requiring students to write about both positive and negative daily experiences without passing judgment
**Seventh session**	Check homework, do complete mindfulness including repeating sessions 4, 5, and 6 each for 20 minutes, presenting the tasks of the next session (recording homework report, breathing space exercise, body examination exercise, and meditation practice)
**Eighth Session**	Performing body examination exercises, reviewing homework and the whole program

**Repetitive transracial magnetic stimulation (rTMS): **Therapeutic agents were applied per the International Policy for the Optimal Use of TMS (1996) (53) in the left dorsolateral prefrontal cortex, areas 9 and 46 of Brodmann. Our rTMS protocol (10 Hz, 110% RMT, 60 trains of 5 s, 25 s between trains, in total 3000 biphasic pulses in 30 min) fulfilled the current international safety guidelines (54). It should be noted that the subjects received the rTMS treatment following the third session of cognitive therapy for mindfulness. 15 minutes of mindfulness exercises were performed before rTMS sessions.

**Statistical analysis: **Visual and graphical analyses were used to assess the effectiveness of the interventions following the executed plan. Additionally, it was used to analyze the recovery rate of results and the effect size. If at least 50% of patients recover, the results are considered clinically significant (55). Additionally, effect sizes of 0.41, 1.15, and 2.70 have been proposed as D Cohen criteria for effective interventions in a single-case experimental design for low, middle, and high use, respectively (56).

## Results

The demographic characteristics of the subjects are presented in table 2. The contents of table 3 showed that the mean and level of information processing scores of all five patients in the treatment and follow-up stages decreased compared to the baseline stage. Scores from this scale show that patients achieved an overall recovery of 67.24% after the intervention stage and 53.64% after follow-up. Based on the above table results, the first patient in the last treatment session achieved a recovery of 53.30%, and after follow-up, a recovery of 47.02%. The second patient achieved 45.41% recovery after the intervention and 39.43% recovery after follow-up, and the third patient achieved 67.01% recovery after the intervention stage and 59.78% recovery after follow-up. The fourth patient achieved 51.26% recovery after the intervention and 45.74% recovery after follow-up. The fifth patient reached 51.23% recovery after treatment and 37.37% recovery after follow-up. 

**Figure 1:**
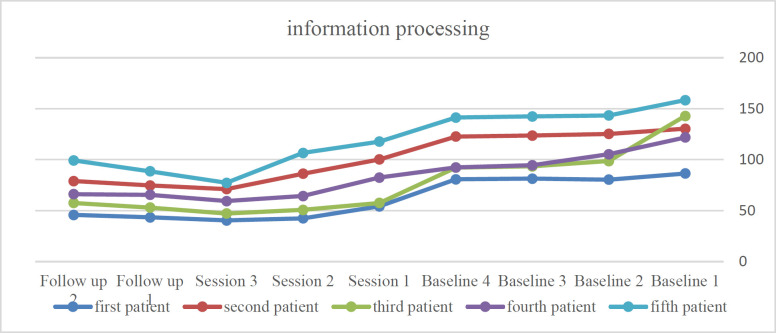
The process of changing information processing (seconds) in the baseline, treatment, and follow-up stages

Evaluation of treatment recovery rate showed that treatment is clinically significant for subjects 1, 3, 4, and 5. Also, the effect size for the first to fifth subjects was 1.83, 1.63, 1.71, 1.61, and 1.62, respectively, which shows that the effect size is middle for all 5 subjects. According to figure 1, the time required to complete the information processing test had decreased for all five subjects, indicating that the subjects' information processing speed has increased, as indicated by the slope of the graphs.

**Table 2:** Demographic characteristics of the subjects

Patients	Age	Gender	Education Level	Marital status	Occupation
**First patient**	29	Male	Diploma	Single	Self-employment
**Second patient**	42	Female	Bachelor	Married	Teacher
**Third patient**	28	Female	Bachelor	Married	Housewife
**Fourth patient**	27	Female	Diploma	Single	Unemployed
**Fifth patient**	42	Female	Diploma	Married	Housewife

**Table 3:** Percentage of information processing recovery (seconds)

Stages of intervention / patients	first patient	Second patient	Third patient	fourth patient	Fifth patient
**Baseline 1**	86.42	130**.**32	142.85	121.84	158.41
**Baseline 2**	80.82	125.28	98.57	105.3	143.35
**Baseline 3**	81.34	123.67	93.44	94.5	142.41
**Baseline 4**	80.74	122.69	92.26	92.33	141.3
**Mean baseline**	82.33	125.49	106.78	103.493	146.368
**Session 1 treatment**	54.18	100.23	57.49	82.4	117.68
**Session 2 treatment**	42.44	86.15	50.72	64.29	106.56
**Session 3 treatment**	40.35	71.14	47.12	59.38	77.25
**Mean course of treatment**	45.6567	85.84	51.7767	68.69	100.497
**Follow up 1**	43.39	74.63	52.95	65.44	88.56
**Follow up 2**	45.78	78.93	57.45	66.11	99.21
**Mean follow-up period**	44.58	76.78	55.2	65.77	93.88
**Percentage of treatment improvement**	53.30	45.41	67.01	51.26	51.23
**Percentage of follow-up improvement**	47.02	39.43	59.78	45.74	37.37
**Effect size**	1.83	1.63	1.71	1.61	1.62

**Table 4:** Percentage of working memory recovery

Stages of intervention / patients	first patient	Second patient	Third patient	Fourth patient	Fifth patient
**Baseline 1**	28	33	50	33	28
**Baseline 2**	32	40	40	33	33
**Baseline 3**	40	40	50	40	37
**Baseline 4**	40	40	50	40	36
**Mean baseline**	35	38.25	47.5	36.5	33.5
**Session 1 treatment**	66	50	66	44	50
**Session 2 treatment**	66	60	75	46	62
**Session 3 treatment**	71	66	80	57	66
**Mean course of treatment**	67.667	58.667	73.667	49	59.333
**Follow-up 1**	62	62	66	55	66
**Follow -up 2**	60	57	66	50	60
**Mean follow-up period**	61	59.5	66	52.5	63
**Percentage of treatment improvement**	*153.6	*100	*60	*72.7	*135.7
**Percentage of follow-up improvement**	*114.3	*72.7	*32	*51.5	*114.3
**Effect size**	1.95	1.71	1.95	1.43	1.68

An asterisk (*) indicates that the changes as being incremental.

**Figure 2:**
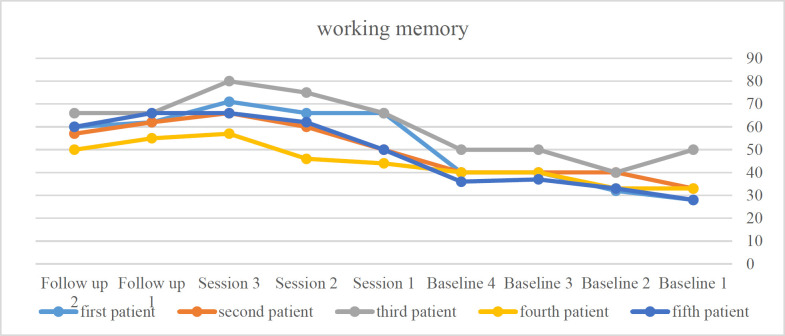
The process of changing working memory in the baseline, treatment, and follow-up stages

The contents of table 4 showed that the mean and level of working memory scores of all five patients in the treatment and follow-up stages increased compared to the baseline stage. The scores obtained from this scale showed that patients achieved an overall recovery of 104.04% after the intervention stage and 76.98% after follow-up. Based on the above table results, the first patient in the last treatment session achieved a recovery of 153.6%, and after follow-up, a recovery of 114.3%. The second patient achieved 100% recovery after the intervention and 72.7% recovery after follow-up. The third patient achieved 60% recovery in the after-intervention stage and 32% recovery after follow-up. The fourth patient achieved 72.7% recovery after the intervention and 51.5% recovery after follow-up. The fifth patient has reached 135.7% recovery after treatment and 114.3% recovery after follow-up. Since the percentage of subjects who improved as a result of the treatment is greater than 50, the treatment is clinically significant for all five subjects, and their working memory improves. Also, the effect size for the first to fifth subjects was 1.95, 1.71, 1.95, 1.43, and 1.68, respectively, which shows that the effect size is middle for all 5 subjects. Working memory improved in all five subjects, as indicated by the slope of the graphs in figure 2.

## Discussion

This study aimed to evaluate the effectiveness of mindfulness-based cognitive therapy combined with rTMS on the information processing and working memory of MS patients. The current study's findings indicated that MS patients' information processing improved following mindfulness-based cognitive therapy with rTMS. These results are consistent with the results of van Leeuwen, Singer & Melloni (57), Manglani et al. (58), Guse, Falkai, Wobrock (59). Manglani et al. (58) in a study on people with multiple sclerosis with used Symbol Digit Modalities Test (SDMT) showed that in people with multiple sclerosis, a 4-week of mindfulness meditation training improves processing speed and goes beyond adaptive computer cognition and waiting list training. Moreover, mindfulness redirects attention away from unread thoughts and toward voluntary concentration. In various situations, the individual can avoid secondary processing of thoughts, feelings, and bodily sensations evoked by the schemas, and the individual's total cognitive capacity is used to perform the task more effectively (60). 

Additionally, mindfulness-based interventions can improve a patient's cognition by causing structural changes in the brain (61). Furthermore, it has been shown that rTMS can alter the structural, functional, and molecular properties of neurons, which may depend on the simultaneous transfer of action potentials (34-35). Evidence for changes caused by rTMS is due to cerebral blood flow, glucose metabolism, and neuronal excitability in the brain's stimulated region and junctional areas (62). Also, it has been shown that meditation, plus increasing cortical blood flow, causes functional changes that affect structural changes in the brain. For example, it increases the thickness of many structures in the brain, especially the prefrontal cortex and areas of the anterior cingulate cortex, the right anterior insula, and the right, middle, and superior frontal sulcus (63). Therefore, cognitive therapy of mindfulness combined with rTMS increases the focus of attention through structural changes and increased cerebral blood flow, which improves the speed of information processing in MS patients.

Moreover, the present study results showed that the working memory of MS patients improved after mindfulness-based cognitive therapy with rTMS. This finding is consistent with the research of Youngs et al. (64), Brunoni, Vanderhasselt (65), Kedzior et al. (66). Hulst et al. (2017) in a study examined the effectiveness rTMS on working memory performance, brain activation and functional connectivity in MS patients. In this study, 17 MS patients and 11 healthy controls were assessed by working memory test (n-back) in underwent 3 experimental sessions (baseline, real-rTMS, sham-rTMS). The results of their study showed that the performance of patients in the working memory test (n-back) improved after real-rTMS compared to baseline. While this improvement was not observed in the sham-rTMS group compared to the baseline. (67). Neuroimaging studies have demonstrated that regular mindfulness and meditation exercises result in functional and structural changes in areas of the brain associated with learning, memory, attention, and emotion regulation (68-69). Also, meditation techniques have been shown to increase the output of the vagal nerve, which reduces heart rate and respiration while increasing the sedative response (68-69). Eysenck et al. (70) suggested that the control aspect of central executive attention is impaired by anxiety. In particular, the central executive inhibition function can no longer effectively divert attention from the work of irrelevant stimuli. Due to the success of memory tasks in directing attention to information relevant to a goal, a link between attention control and memory has been established (71). Researchers have suggested that the processes of attention and memory are close forms of cognitive control, and both are likely to be influenced by mindfulness meditation (30). Additionally, a meta-analysis of studies involving repetitive transracial magnetic stimulation of the dorsolateral prefrontal cortex confirmed a significant improvement in working memory accuracy and reaction time measured by the n-back test (65). Simultaneous performance of a cognitive task during stimulation can potentially increase the effects compared to stimulation alone (72). Consistent with this hypothesis, the results of a recent study have indicated that rTMS combined with the concurrent performance of a cognitive task improves cognitive function in Alzheimer's patients (73). Therefore, it is not far-fetched that rTMS combined with mindfulness techniques will improve the working memory in MS patients. The small number of subjects studied and sampling limited to Imam Khomeini Hospital in Tehran can reduce the generalization of results. Also, there are limitations to the case study, although the single-case experimental design affects each participant, internal and external validity are still concerns. As a result, it is recommended that future research employs experimental designs with a control group.
